# Diffusion Tensor Imaging of Rat Rotator Cuff Muscle with Histopathological Correlation: An Exploratory Study

**DOI:** 10.21203/rs.3.rs-4791101/v1

**Published:** 2024-09-03

**Authors:** James Lo, David B. Berry, Qingbo Tang, Xin Cheng, Marco Toto-Brocchi, Jiang Du, Samuel R. Ward, Yajun Ma, Eric Y. Chang

**Affiliations:** University of California, San Diego; University of California, San Diego; University of California, San Diego; University of California, San Diego; University of California, San Diego; University of California, San Diego; University of California, San Diego; University of California, San Diego; VA San Diego Healthcare System

**Keywords:** diffusion tensor imaging, rotator cuff, skeletal muscle, histological correlation

## Abstract

Diffusion tensor imaging (DTI) is a magnetic resonance imaging (MRI) technique that can be used to assess microstructural features of skeletal muscle that are related to tissue function. Although widely used, direct correlations between DTI derived metrics such as fractional anisotropy and spatially matched tissue microstructure assessed with histology have not been performed. This study investigated the relationship between scalar-based DTI measurements and histologically derived muscle microstructural measurements in rat rotator cuff muscles. Despite meticulous co-localization of MRI and histology data, negligible correlations were found between DTI metrics and histological measurements including muscle fiber diameter, cross-sectional area, and surface-to-volume ratio. These findings highlight the challenges in validating DTI with histology due to requirements in anatomical co-localization, necessity of high-quality histology, and consideration of diffusion measurement scales. Our findings underscore the need for further research with optimized imaging parameters to enhance our knowledge regarding the sensitivity of DTI to important features of muscle microstructure.

## Introduction

Diffusion magnetic resonance imaging (dMRI) is an imaging technique that exploits the anisotropic motion of water molecules to generate image contrast, enabling quantification of the restricted diffusion profile of a tissue^1^. It is well-established that dMRI provides unique clues to the microstructure and architecture of tissues that have biological and clinical relevance^2^. Although most widely utilized in neuroimaging, dMRI has been increasingly used for musculoskeletal applications, including skeletal muscle imaging^3^.

dMRI data can be mathematically modeled to fully characterize how diffusion in space varies according to direction, a technique referred to as diffusion tensor imaging (DTI)^2^. One of the goals of DTI applications in skeletal muscle is to assess key features of muscle microstructure related to function including muscle fiber size. Muscle fiber diameter and cross-sectional area are directly related to the isometric force-generating capacity of a muscle fiber^4^ and thus, a noninvasive tool that can measure changes in fiber size associated with injury, pathology, or treatment holds great clinical value. DTI in skeletal muscle has been applied to study clinically relevant applications including assessment of severity of muscular dystrophy^5–8^, inflammatory myositis^9–11^, and denervation^12,13^, as well as age-related muscle changes^14,15^, monitoring recovery after injury^16^, and prediction of return to sport^17^.

Despite these successes, key questions on a more fundamental level remain. For instance, the ability of DTI to resolve specific features of muscle microstructure is unknown. Validation with histology is required for an answer to this question, and the studies to date using histological tissue have provided conflicting results. A few studies have utilized the random permeable barrier model (RPBM), which leverages diffusion time (Δ) dependent stimulated echo DTI data into a mathematical framework used to relate diffusion measurements in tissue to discrete microstructural features of muscle such as fiber size^18^. Some have found no significant associations between these DTI-based features of muscle microstructure and muscle biopsy data, including fiber diameters^15,19^. This has been hypothesized to be due to insufficient anatomic co-localization between modalities; it is highly difficult to precisely colocalize biopsy measurements (< 1 mL in volume)with whole muscle DTI based measurements, especially in large human muscles (hundreds to thousands of mLs in volume). In contrast, systematic simulations of DTI data and histology informed models of skeletal muscle injury indicate that significant relationships should exist, with the strength of the correlation dependent on the specific pulse sequence parameters and mathematical model used for the data^20–22^.

Another important parameter is spatial resolution of the imaging sequence, which is one of the most important practical considerations of any MRI protocol. dMRI/DTI is sensitive to molecular motion on a micrometer scale, but imaging protocols are often on a millimeter scale. Since a muscle fiber is approximately 30–70 μm in diameter, hundreds to tens of thousands of muscle fibers can pass through a single voxel. Thus, the resulting diffusion signal is sensitive to macroscopic anisotropy of the underlying muscle tissue and is not sensitive to heterogeneity within the voxel. It has been suggested that diffusion measurements remain stable among different voxel sizes in phantoms and rat brain tissue^23^, but the effects of imaging resolution on correlations between DTI measures and histologically-derived measurements remains unknown.

The purpose of this exploratory study was to quantify the relationship between scalar-based measures of DTI and histologically-derived microstructural measurements in precisely co-localized rat rotator cuff muscle tissue and to compare the results when imaged at 0.25 mm and 0.5 mm isotropic resolutions.

## Methods and Materials

### Ethical Approval

All methods were performed in accordance with the relevant guidelines and regulations. All animal research was approved by the VA San Diego Healthcare System Institutional Animal Care and Use Committee, with protocol number: A20–005. The reporting in this manuscript follows the recommendations in the ARRIVE guidelines (PLoS Bio 8(6), e1000412, 2010).

### Animals and Rotator Cuff Tear Model

Experiments were performed on Four Lewis rats, which were originally purchased from Charles River Laboratories (Portage, MI, USA). A rat model of chronic massive rotator cuff tearing was adapted for the purpose of increasing the range of myofiber sizes^24^. Specifically, smaller sizes are seen post-injury^24,25^. At 13-weeks of age, supraspinatus and infraspinatus tenotomies were performed on the right shoulders of each animal and the distal 2–3 mm of the tendons were removed. A thin, L-shaped silicone (polydimethylsiloxane [PDMS]) implant was placed over the greater tuberosity and firmly fixed with a size 2–0 nonabsorbable polyester suture to prevent spontaneous tendon healing. After closure, rats were allowed unrestricted cage activity. At 20-weeks post injury, the rats were deeply anesthetized with isoflurane and euthanized via terminal perfusion with 250 ml saline and 300 ml 4% paraformaldehyde (PFA) in phosphate buffer. Each scapula containing all rotator cuff muscles was individually dissected and postfixed in PFA at 4°C for 48 hours.

### Magnetic Resonance Imaging

All scapulae were individually imaged using a 30 mm receive-only surface coil and 82 mm volume coil for transmission on a 3T pre-clinical MRI scanner (BioSpec 3T, Bruker, Billerica, MA, USA). Relaxometry was performed on two randomly selected fixed rotator cuff muscle samples using the Image Sequence Analysis tool on ParaVision 360 v3.3 (Bruker) to inform the optimal parameters for the DTI sequences. Using a variable repetition time (TR) rapid acquisition with relaxation enhancement sequence with 5 TRs ranging from 850–4000 ms, mean mono-exponential T1 was approximately 900 ms. For optimal SNR efficiency (~1.3x T1^26^), a TR of 1200 was selected for the DTI sequences. Using a Carr−Purcell−Meiboom−Gill sequence with TR = 7500 ms and 8 echo times (TEs) ranging from 9–72 ms was, mean mono-exponential T2 was 35 ms. A TE of 23–25 ms was selected as for the DTI sequences since approximately 50% of the transverse signal would remain based on the T2 decay.

Two sets of spin echo single-pulsed field gradient (sPFG) DTI sequences with fat-suppressed spin-echo readout were employed, one with 0.25 mm isotropic resolution and the other with 0.5 mm isotropic resolution. The 0.25 mm isotropic sequence included the following imaging parameters: TR/TE = 1200/25 ms, number of excitations (NEX) = 2, bandwidth = 30 kHz, 30 diffusion encoding directions with b = 500 s/mm^2^ (gradient duration/gradient separation = 3.1/15 ms), five b = 0 s/mm^2^ images, with a total imaging time of approximately 80 hours. The 0.5 mm isotropic sequences included the following imaging parameters: TR/TE = 1200/23 ms, NEX = 1, bandwidth = 30 kHz, 30 diffusion encoding directions with b = 500 s/mm^2^ (gradient duration/gradient separation = 3.1/15 ms), five b = 0 s/mm^2^ images, with a total imaging time of approximately 10 hours.

### Magnetic Resonance Imaging Data Analysis

The supraspinatus and infraspinatus muscles were manually segmented from the 0.25mm isotropic dMRI b = 0 s/mm^2^ images using Horos (v4.0, Purview, Annapolis, MD, USA). Segmented volumes were scaled to the 0.5mm resolution using the analysis of functional neuroimaging (AFNI) command 3dresample^27,28^. Diffusion weighted images were denoised using a local principal component analysis filter^29^. The diffusion tensor was calculated using the AFNI command 3dDWItoDT and fractional anisotropy (FA), mean diffusivity (MD), and radial diffusivity (RD) were the parameters that were included in the analysis.

### Histological Preparation and Acquisition

Samples were decalcified in 20% ethylenediaminetetraacetic acid (EDTA) for 4 weeks with a medium change every 72 hours that was checked weekly with radiography. Following decalcification, the samples were copiously washed in phosphate buffered saline, treated with 30% sucrose for cryoprotection, snap-frozen in liquid nitrogen-cooled isopentane, and then embedded in optimal cutting temperature (OCT) compound.

Sectioning and staining performed by a subspecialized MSK pathologist (X.C., with 24 years of experience). Twelve μm-thick cryosections were collected using a cryostat microtome in the transverse direction relative to the spine and body of the scapula, corresponding to the “sagittal oblique” plane on human MRI. At least three locations were sectioned: medial scapular, mid-scapular in the central region of the muscle belly, and myotendinous junction near the lateral margin where the scapular spine and body merged. All sections were stained with hematoxylin and eosin (H&E) and Masson trichrome (Epredia Richard-Allan Scientific Masson Trichrome Kit, 22110648, Fisher Scientific, Hampton, NH, USA). Brightfield image digitization was performed using a slide scanner at 0.22 μm/pixel (AxioScan.Z1, ZEISS, Thornwood, NY, USA).

### Histologic Image Analysis

Quality control (QC) was performed on each digitized histology slide with the following requirements for inclusion in the study: scapular bone must be entirely preserved to permit co-localization with MRI, and at least 50% of either the supraspinatus or infraspinatus muscle must be preserved without tissue loss, folds, or distortion artifacts.

Whole-muscle myofiber boundary segmentation was performed using HALO AI (v3.6.4134, Indica Labs, Albuquerque, NM, USA). Specifically, the pre-trained Membrane Segmentation network feature in the software was fined-tuned using at least 50 manually segmented myofibers per slide by an MSK pathologist (X.C.). Each slide employed its own fine-tuned algorithm and QC was double checked in consensus by a MSK pathologist (X.C.) and MSK radiologist (E.Y.C.). Tendon regions and artifacts were manually excluded.

MATLAB software (R2024a, Mathworks Inc., Natick, MA) was used for size measurements on the histologic slides and processing of MRI data. Myofiber diameter, cross-sectional area, and surface area to volume (S/V) ratio measurements were determined from the whole-muscle segmentations. While myofiber diameter and cross-sectional area are commonly assessed features of muscle microstructure related to function, S/V is a relatively understudied feature of muscle microstructure that may have high correlations with DTI metrics, including RPBM modeling or diffusion tensor subspace imaging analysis^22^. Thus, while not a standard muscle microstructural measurement, as its relationship to function is still relatively unknown, this feature was additionally included as a biomarker for histologic assessment. Artifactual gaps between myofibers are expected as part of the histological preparation process and this was compensated through down-sampling of the spatial resolution of the histological slides using an averaging filter.

MRI data was reviewed by a MSK radiologist (E.Y.C., with 13 years of experience) and co-localization with the histological section locations was meticulously performed based on scapular bone and muscle dimensions. Signal-to-noise (SNR) was measured for all included samples at each resolution, defined as mean signal over the muscle divided by the standard deviation of the noise. Non-rigid body registration of muscle boundaries between the DTI and histology maps was performed. Further down-sampling was performed on these co-registered histological sections and the 0.25 mm isotropic resolution DTI data (2×2 kernel) to match the dimensions of the 0.5 mm isotropic resolution DTI data. Histograms were reviewed during each down-sampling step to ensure that the measurements and distributions remained consistent.

### Statistical analyses

Scatter plots were generated on a pixel-by-pixel basis from co-localized DTI and histology data. Pearson’s correlations were performed between each scalar-based DTI metric (MD, RD, and FA) and each histologic metric (diameter, area, S/V) for all included samples and interpreted as: 0–0.29, negligible; 0.3–0.49, low; 0.5–0.69, moderate; 0.7–0.89, high; 0.9–1.0, very high^30^. Due to the exploratory nature of this study utilizing a very small sample size, adjustments for internal dependence and multiple comparisons were not made. Thus, all results are reported only informally and will need to be confirmed with independent studies. Statistical analysis was performed in MATLAB.

## Results

Out of 48 possible muscle locations (4 rats, 2 shoulders per rat, each containing both supraspinatus and infraspinatus muscles, with 3 locations per muscle), 26 did not pass histologic QC. After QC exclusions, data from 2 rats remained (4 shoulders with 8 rotator cuff muscles) in the form of 22 distinct muscle locations, which were included in this study (Supplemental Figure 1).

Mean SNR of the rotator cuff muscles was: 92 on the b = 0 s/mm^2^ 0.25 mm isotropic images, 46 on the b = 500 s/mm^2^ 0.25 mm isotropic images, 156 on the b = 0 s/mm^2^ 0.5 mm isotropic images, and 76 on the b = 500 s/mm^2^ 0.5 mm isotropic images.

**Figure 1:**
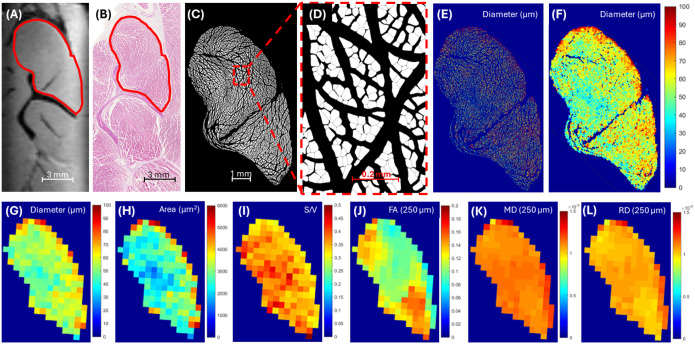
Representative example of the supraspinatus muscle. 3D spin-echo fat-saturated DTI image at 0.25 mm isotropic resolution (A) and the corresponding histological section after H&E staining (B) with the supraspinatus muscle outlined in red. Automated whole-muscle myofiber boundary segmented images at low (C) and high (D) magnification. Automated fiber size (diameter) color map before (E) and after (F) 100x down-sampling of spatial resolution shows that the majority of the gaps introduced from histological preparation are omitted. After manual co-registration to the DTI images and further down-sampling to match the resolutions, diameter, area, and surface-to-volume (S/V) ratio pixel maps are generated (G-I). Corresponding fractional anisotropy (FA), mean diffusivity (MD), and radial diffusivity (RD) pixel maps at 0.25 mm isotropic resolution are shown (J-L).

**Figure 2:**
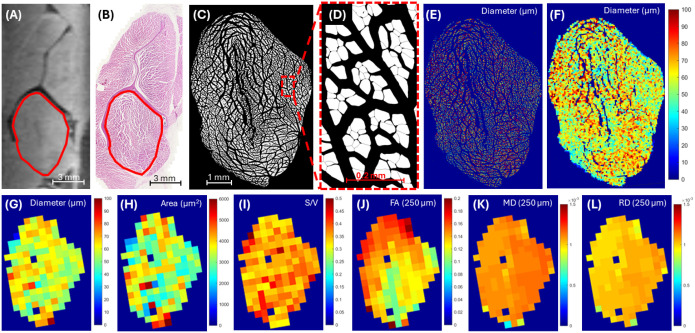
Representative example of the infraspinatus muscle. 3D spin-echo fat-saturated DTI image at 0.25 mm isotropic resolution (A) and the corresponding histological section after H&E staining (B) with the supraspinatus muscle outlined in red. Automated whole-muscle myofiber boundary segmented images at low (C) and high (D) magnification. Automated fiber size (diameter) color map before (E) and after (F) 100x down-sampling of spatial resolution shows that the majority of the gaps introduced from histological preparation are omitted. After manual co-registration to the DTI images and further down-sampling to match the resolutions, diameter, area, and surface-to-volume (S/V) ratio pixel maps are generated (G-I). Corresponding fractional anisotropy (FA), mean diffusivity (MD), and radial diffusivity (RD) pixel maps at 0.25 mm isotropic resolution are shown (J-L).

Figures 1 and 2 show representative examples for two muscle locations, including one in the supraspinatus and one in the infraspinatus, respectively. Guided by the scapular bone landmarks and muscle contours, co-registration between the DTI data (Figures 1A and 2A) and corresponding histological sections (Figures 1B and 2B) for all included samples was successful. Myofiber segmentation (Figures 1C/D and 2C/D) was also successful. Gaps introduced by the histological preparation process were successfully minimized through down-sampling with an averaging filter (Figures 1E/F and 2E/F). The resultant pixel maps of histological measures (Figures 1G–I and 2G–I) after down-sampling were readily correlated with DTI metric pixel maps (Figures 1J–L and 2J–L).

**Figure 3:**
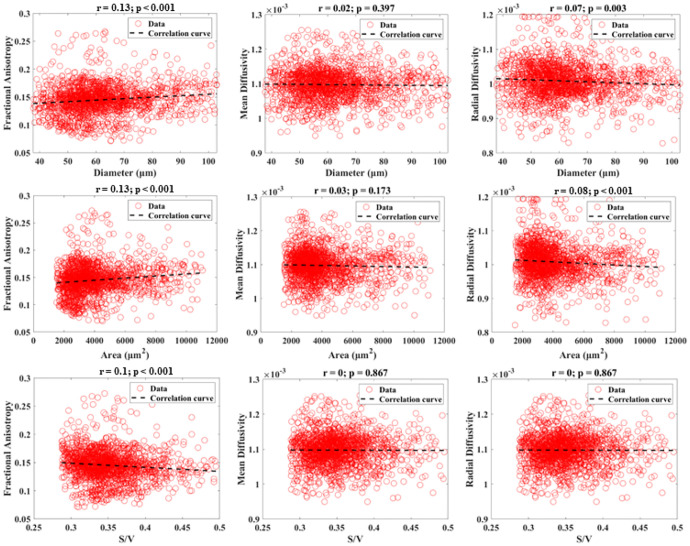
Combined scatter plots showing the relationships between diffusion measurements (FA, MD, and RD) measured from DTI at 0.25 mm isotropic resolution versus histological measurements (diameter, area, and S/V). Pearson correlation results are displayed.

**Figure 4:**
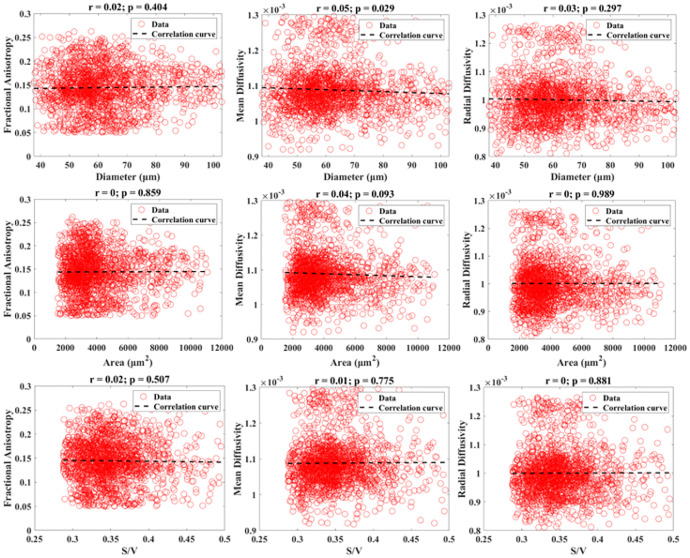
Combined scatter plots showing the relationships between diffusion measurements (FA, MD, and RD) measured from DTI at 0.5 mm isotropic resolution versus histological measurements (diameter, area, and S/V). Pearson correlation results are displayed.

Figures 3 and 4 show the scatter plots and Pearson correlations for the 0.25 mm isotropic resolution and 0.5 mm isotropic resolution DTI data with their corresponding histologic measurements of all included muscle locations. All DTI measures yielded negligible correlations (r=0–0.13) with histologically derived microstructural measurements. No consistent trend was found between the 0.25 mm and 0.5 mm isotropic resolution correlations.

## Discussion

In this study we investigated the relationship between scalar-based measures of DTI, including MD, RD, and FA, and histologically derived microstructural measurements, including myofiber diameter, area, and S/V, in precisely co-localized rat rotator cuff muscle tissue. Unfortunately, we failed to find a substantial relationship between any of the comparisons, for either the 0.25 mm or 0.5 mm isotropic datasets.

To potentially explain our results requires a discussion of some of the variables with DTI, particularly the conditions used in this study, which can be viewed in broad categories relating to the methods of measurement and the conditions of the tissue. We utilized a diffusion time (Δ) of 15 ms, which is in the intermediate range^31^, and typical of that which can be acquired with the spin echo sPFG method for diffusion preparation to study muscle microstructure^3^. Although the most commonly used method for diffusion sensitization, the main drawback to the spin echo SPFG method is that Δ is constrained by the TE (23–25 ms in our protocol). The TE of the sequence must be sufficiently short as muscle is a short T2 tissue (measured at 35 ms in our case), and SNR must be a minimum of 25 for accuracies of the DTI metrics to be within 5% or 50 to be within 1%^32,33^. Δ may also be substantially shorter, but either very high-performance pre-clinical gradients are required or an oscillating gradient spin echo (OGSE) diffusion encoding strategy must be employed. Earlier nuclear magnetic resonance studies found that short Δ values of a few seconds or less were able to probe S/V ratios in porous media^34^ and erythrocytes^35^. OGSE diffusion encoding has also shown promising results for S/V ratio measurements in anisotropic fiber phantoms^36^ and was more recently employed to study human muscle^37^, but the sensitivity of short Δ values to microstructural measurements in muscle remain to be determined.

Longer Δ can be achieved using stimulated echo diffusion preparations. Although SNR may be up to 2.9x lower compared lower compared with spin echo sPFG^38^, Δ values of greater than 1 second can be obtained^39^. Using Monte-Carlo simulations, Berry et al. previously demonstrated that Δ should be ~170 ms to maximally discriminate fiber size if only a single diffusion experiment can be performed, though ideally multiple diffusion times should be sampled^21^. This was independently supported by McDowell et al., where Δ = 190 ms yielded the largest effect size in discriminating between muscles from healthy adult volunteers, boys with Duchenne muscular dystrophy, and age-matched controls^7^. The studies to date using long Δ and RPBM analysis have provided conflicting results regarding the sensitivity of diffusion measures to muscle fiber geometries/sizes. Winters et al. used Δ values ranging from 20–700 ms on mice and found significant correlations between histological perimeter-to-area (P/A) and DTI-RPBM S/V ratios, for both ex vivo (r=0.71) and in vivo (r=0.56) conditions^40^. However, Cameron et al. used Δ values of 100 and 300 ms on muscle biopsy samples from 5 participants^19^ and Malis et al. used 10 Δ values ranging from 15–590 on biopsy samples from 10 participants^15^, with neither being able to demonstrate a statistically significant association between DTI-RPBM measures and fiber sizes. Notably, the authors of these prior studies acknowledge that there were challenges with localizing the same anatomic area between histology and MRI. Thus, this underscores the importance of studies such as ours where great care is taken for anatomic co-localization.

The muscle used in this study was post-mortem, post-fixed, and at room temperature during imaging, each resulting in reductions in the apparent diffusion coefficient compared with the in vivo condition. Sapienza et al. found that the apparent diffusion coefficient of muscle progressively decreased with longer post-mortem intervals^41^. Rats were perfused in our study, so only minimal reductions would be expected. The fixation, however, reduces diffusivity. Although the magnitude is unknown in muscle, decreases from 30–80% are seen in neurological tissue^42,43^. Scanning at room temperature results in a further decrease in apparent diffusion coefficient of approximately 30% compared with live tissue (~2%/°C)^44^. A rough estimate of diffusion length (ℓ_D_) is provided by the square of the product of λ1 and Δ^45^. λ1 is the measurement of diffusion along the length of a muscle fiber and thus, is the closest measurement of unrestricted molecular diffusion that can be performed given this application. λ1 values of ~1.6 mm^2^/s have been reported in normal rat muscles in vivo^46^. Assuming a decrease of 30% due to temperature differences, and a further decrease of 30–80% due to fixation, an expected ℓ_D_ of 2–3 μm would be expected with the intermediate Δ of 15 ms used in our study. This is at least an order of magnitude below the expected muscle fiber diameter and appears to be insufficient to characterize the interactions with and restriction by the sarcolemma.

This study highlights the challenges in directly validating dMRI measurements in real tissue. While these experiments are highly controlled and precise, some concessions in the form of imaging live animals and imaging within a clinically relevant scan time had to be sacrificed. This study supports the use of simulation-based experiments to develop theoretical frameworks between the dMRI signal and underlying microstructure, as it allows the user to precisely control microstructure, SNR, pulse sequence parameters, and diffusion characteristics^20–22,32,33,47–50^. Translating the findings between simulation-based platforms to actual DTI experiments is still a challenge, as in a tissue-based experiment, the ground truth (i.e. microstructural features) is unknown until the tissue is processed and precisely co localized, as was shown in this study. Diffusion phantoms with muscle informed microstructure^51^ provide an excellent balance between these two platforms, as they provide user defined features of microstructure that can be directly measured and validated against a ground truth.

In addition to the aforementioned limitations in tissue quality, a limitation of this study includes low sample size. However, this is an exploratory study and the methodology and findings should inform more optimal parameters and experimental conditions in future studies with greater numbers. Additionally, the myofiber dimensions in our study are smaller than the in vivo condition due to the histological processing. Specifically, the sucrose concentration was hypertonic, resulting in cell shrinkage. However, this should not have affected correlation analysis.

In conclusion, we were unable to find a substantial relationship between scalar-based measures of DTI and histology-derived microstructural measures in muscle using a diffusion time of 15 ms for either 0.25 mm or 0.5 mm isotropic resolution datasets. Our study confirms that the experimental conditions greatly influence the sensitivity to muscle fiber size and intermediate diffusion times used with spin echo sPFG DTI sequences can produce negligible sensitivity to direct measures of muscle tissue microstructure.

## Data Availability

All data generated or analyzed during this study are included in this published article [and its supplementary information files] and available from the corresponding author at reasonable request.
